# Association Between Vitamin D Status and Diabetic Complications in Patients With Type 2 Diabetes Mellitus: A Cross-Sectional Study in Hunan China

**DOI:** 10.3389/fendo.2020.564738

**Published:** 2020-09-16

**Authors:** Ying Xiao, Ling Wei, Xiaofen Xiong, Ming Yang, Lin Sun

**Affiliations:** Department of Nephrology, Hunan Key Laboratory of Kidney Disease and Blood Purification, The Second Xiangya Hospital of Central South University, Changsha, China

**Keywords:** vitamin D deficiency, diabetic retinopathy, diabetic kidney disease, diabetic foot ulcers, cross-sectional study

## Abstract

**Background:** Vitamin D status has been linked to diabetes-related complications due to multiple extraskeletal effects. We aimed to investigate the association between vitamin D deficiency (VDD) and diabetic vascular complications, including diabetic retinopathy (DR), diabetic kidney disease (DKD), and diabetic foot ulcers (DFU).

**Methods:** A total of 4,284 Chinese patients with type 2 diabetic mellitus (T2DM) were enrolled into the cross-sectional study. VDD was defined as serum 25-hydroxyvitamin D <50 nmol/L. Demographic data, physical measurements, laboratory measurements, comorbidities, and related medications were collected and analyzed by VDD status. Poisson regression with robust variance estimation and binary logistic regression were performed to explore the relationship between VDD and diabetic complications.

**Results:** The prevalence of VDD, DR, DKD, DFU accounted to 71.7% (95% confidence intervals [CI]: 70.3-73.0%), 28.5% (95% CI: 27.2-29.9%), 28.2% (95% CI: 26.8-29.5%), and 5.7% (95% CI: 5.1-6.5%), respectively. The prevalence ratios (95% CI) for DR and DKD by VDD status, adjusted for demographics, physical measurements, laboratory measurements, related complications, and comorbidities, and medications, were 1.093 (0.983-1.215) and 1.041 (0.937-1.156), respectively. The odds ratio (95% CI) for DFU by VDD status was 1.656 (1.159-2.367) in the final adjusted model. Meanwhile, the prevalence of VDD was significantly higher in patients with DFU compared with patients without DFU.

**Conclusions:** The present study firstly indicated that VDD was significantly associated with a higher prevalence of DFU among Chinese T2DM patients. The association between VDD status and DR or DKD was not significant when adjusting for all potential covariates. Vitamin D screening or supplementation may be beneficial to prevent DFU and improve the prognosis of T2DM patients.

## Introduction

Diabetes mellitus is a severe and growing public health problem with a substantial economic burden worldwide. It is estimated that 463 million people are living with diabetes in 2019, and this estimate is projected to rise to 700 million by 2045 without urgent and sufficient actions (1). In China, ~11% of the population has diabetes, with a significant proportion remaining undiagnosed (2). More than 90% of diabetes mellitus are type 2 diabetes mellitus (T2DM). The escalating epidemic of T2DM can be attributed to aging, the rise in obesity, sedentary lifestyles and energy-dense diets (3, 4). T2DM can lead to severe microvascular and macrovascular complications, including diabetic retinopathy (DR), diabetic kidney disease (DKD), and diabetic foot ulcers (DFU) (5).

Generally, DR is the main cause of preventable blindness globally, with a prevalence of ~34.6% (6). The prevalence of DKD varies from 20 to 40% in patients with diabetes (7) and it has been the leading cause of end-stage renal disease in Chinese hospitalized patients since 2011 (8). Besides, DFU is responsible for the high numbers of lower-limb amputations and increased risk of mortality of diabetic patients (9). The prevalence of DFU is 4%-10% and the lifetime incidence has been estimated to be 10–25% among persons with diabetes (10, 11). All these complications lead to disability, reduce the quality of life, and impair economic development (5). Therefore, it is of great significance to identify key modifiable factors associated with these complications so as to improve the prognosis of T2DM.

Vitamin D, a pleiotropic steroid hormone, can exert various effects through binding to its specific receptor-vitamin D receptor. In addition to mediating bone metabolism by regulating calcium and phosphorus homeostasis, vitamin D also modulates cell proliferation, differentiation, apoptosis, immune function, inflammation response, as well as vascular and metabolic properties (e.g., insulin secretion and insulin sensitivity) (12–14). In the past few years, the association between vitamin D deficiency (VDD) and other nonclassical outcomes besides skeletal disorders has drawn increasing attention, especially diabetes and diabetes-related complications (14–17). The inverse association between vitamin D levels and risk of DR (18), DKD (19), or DFU (20) among diverse populations has been demonstrated, whereas other studies reported discordant results regarding the correlations between vitamin D status and these three complications of diabetes (21–23). More importantly, large-scale epidemiological studies on the association of VDD and diabetes-related complications among the Chinese population are scarce (24, 25), which have revealed that lower vitamin D level was associated with increased albuminuria/creatine ratio and higher risk of DR. And no study has evaluated the relationship between VDD and DFU in Chinese diabetic population.

In this cross-sectional study, we aimed to explore the prevalence of VDD, and address the associations between VDD and three severe vascular diabetic complications (i.e., DR, DKD, DFU) in a Chinese T2DM population retrospectively.

## Materials and Methods

### Study Population

Participants we enrolled were admitted to the Department of Metabolism and Endocrinology and Diabetes Center of the Second Xiangya Hospital of Central South University from January 2014 to July 2018. Only inpatients aged ≥18 years with a definite diagnosis of T2DM were included in the study. T2DM was defined according to the American Diabetes Association classification (26). The exclusion criteria were as follows: (1) with missing serum 25-hydroxyvitamin D (25[OH]D) data; (2) pregnant or lactating females; (3) with a known diagnosis of nephrolithiasis, glomerular or lupus nephritis, primary nephrotic syndrome, or other identified kidney diseases; (4) serum parathormone >6.9 pmol/L or <1.6 pmol/L; (5) serum calcium <2.1 mmol/L or serum phosphorus >1.6 mmol/L; (6) estimated glomerular filtration rate (eGFR) <15 mL/min/1.73 m^2^ or missing, which was calculated using the abbreviated Modification of Diet in Renal Disease (MDRD) equation: 186×(serum creatinine)^−1.154^×(age)^−0.203^×(0.742 if female) (27). A total of 4,284 participants were included in this cross-sectional study and their clinical data were extracted from the electronic medical record system. This study was complied with the Declaration of Helsinki and was approved by the ethics committee of the Second Xiangya Hospital of Central South University.

### Data Collection

General demographic information, including age, sex, smoking and drinking status, duration of diabetes, and family history of diabetes were collected. Physical examination (including body weight and height, blood pressure) was performed by professional caregivers. Body mass index (BMI) was calculated as weight divided by height squared and waist-hip ratio was computed as the waist circumference divided by the hip circumference.

Fasting blood sample and 24-h urine sample were obtained from each participant for further biochemical analysis. The laboratory measurements collected in this study included: serum 25(OH)D, albumin, triglycerides, total cholesterol, high-density lipoprotein cholesterol (HDL-C), low-density lipoprotein cholesterol (LDL-C), fasting plasma glucose, glycated hemoglobin (HbA1c), serum calcium, serum phosphorus, serum uric acid, serum creatinine, and 24-h urine albumin (24HUALB). Notably, serum 25(OH)D concentration was determined by chemiluminescence assay (Siemens ADVIA Centaur XP, Germany) and the detection limit was <10.5 nmol/L. When 25 (OH) D was lower than the detection limit and was treated as a continuous variable, a value of 10.5 nmol/L was used. The Homeostasis Model Assessment 2-insulin resistance (HOMA2-IR) was calculated with the HOMA2 calculator [https://www.dtu.ox.ac.uk/homacalculator/ (updated 2013)].

In addition, diabetic complications (i.e., DR, DKD, DFU, diabetic peripheral neuropathy [DPN]) and related comorbidities (i.e., hypertension, dyslipidemia, coronary heart disease, cerebrovascular disease) were also evaluated. The medication of participants included blood pressure-lowering therapy (BPLT) (use of angiotensin converting enzyme inhibitors [ACEIs] or angiotensin II receptor blockers [ARBs]), lipid-lowering therapy (LLT) (statins) and glucose-lowering therapy (GLT). GLT was divided into four categories (i.e., no medication, oral hypoglycemic agents [OHA] only, insulin only, or using OHA plus insulin).

### Definition

VDD was defined as serum 25(OH)D <50 nmol/L (20 ng/mL). Conversely, the 25(OH)D level of the no VDD group was ≥50 nmmol/L. The presence of DR was confirmed by a professional ophthalmologist using dilated fundoscopy according to the definition of the Global Diabetic Retinopathy Project Group (28). DKD was defined mainly based on albuminuria and a decline of eGFR (<60 mL/min/1.73 m^2^), which was not caused by other causes than diabetes. DFU was mainly defined according to diabetic foot problems, such as ulceration, infection, ischemia, gangrene, or even amputation. DPN was diagnosed by analyzing clinical symptoms (e.g., sensory loss, pain, muscle weakness etc.), neurologic examinations and the results of nerve conduction tests (29). Hypertension was defined when blood pressure was ≥ 140/90 mmHg on three separate occasions after hospital admission by physicians, a prior diagnosis of hypertension or taking antihypertensive drugs. Coronary heart disease and cerebrovascular disease were defined as self-reported history of these diseases, respectively, regardless of disease severity. The definition of dyslipidemia was as follows: total cholesterol ≥6.22 mmol/L, triglycerides ≥2.26 mmol/L, LDL-C ≥ 4.14 mmol/L, HDL-C< 1.04 mmol/L.

Additionally, subjects were divided into three groups by age (i.e., aged 18–44, young adults; aged 45–64, middle age adults; elderly, aged ≥65 years). Participants were also categorized into four groups based on the levels of BMI according to BMI criteria established by the Working Group on Obesity in China (WGOC) (30): underweight (<18.5 kg/m^2^), normal weight (18.5–23.9 kg/m^2^), overweight (24.0–27.9 kg/m^2^), and obese (≥28.0 kg/m^2^). Glycemic control was classified based on HbA1c levels as either good (<7%) or poor (≥7%). The level of serum uric acid was defined as normal (<420 μmol/L) and high (≥ 420 μmol/L).

### Statistical Analysis

Normally distributed continuous variables were presented as the mean ± standard deviation and compared by Student's *t* test. The Mann-Whitney test was used for non-normally distributed continuous variables, which were reported as median and interquartile range (25–75%). Categorical variables were summarized by frequency counts with percentages, and the chi-square test was performed to evaluate differences between groups. For continuous variables with missing values <5%, the missing values were replaced by the mean value of the corresponding variable. Two-tailed *P*-values < 0.05 were considered statistically significant.

In regression analyses, a total of 4,176 participants without missing value in smoking status, drinking status and family history of diabetes were included. HOMA2-IR (19.7% missing) and 24HUALB (9.0% missing) were analyzed by creating a dummy variable corresponding to missing values, respectively. As prevalence of DR and DKD in T2DM patients were not rare, Poisson regression with robust variance estimation were conducted instead of logistic regression to directly estimate the prevalence ratios (PR), along with 95% confidence intervals (CI), and to avoid the overestimation of risk ratios by odds ratio (31). The association between VDD status and DFU was still analyzed using binary logistic regression. Potential confounders (age, sex, duration of diabetes, smoking status, drinking status, BMI, and waist-hip ratio) and the candidate variables with a *P* value < 0.1 on univariate analysis (data not shown) were all included in the multivariable model to analyze the relationship between VDD status and diabetic complications of T2DM (i.e., DR, DKD, and DFU).

The SPSS software (version 25.0; IBM Corp., Armonk, NY) and Stata software (version 14.0; Stata Corp., College Station, TX) were used for statistical analysis. Graphing were performed using Graphpad Prism 7 software (Graphpad Prism Software Inc., La Jolla, CA).

## Results

### Baseline Characteristics of the T2DM Study Population

Table 1 displays the descriptive characteristics of this T2DM study population, both overall and stratified by VDD status. A total of 4284 participants were analyzed in this study. Slightly more than half (52.6%) were male and middle age adults (aged 45–64 years) made up 57.0% of the population. The proportion of participants was similar between groups with different duration of diabetes (33.5, 34.4, and 32.1%). By our primary definition, poor glycemic control, dyslipidemia and hypertension was observed in 82.1, 64.7, and 54.0% of subjects. The proportion of patients undergoing BPLT was 41.8% and patients received LLT accounted for 73.9%. Besides, the vast majority (97.3%) were on GLT, including insulin and/or oral hypoglycemic drugs.

**Table 1:** Baseline characteristics among participants, overall and by VDD status.

	**Overall**	**With VDD**	**Without VDD**	***P* value**
*N*	4284	3071	1213	
**Demographics**				
Age, *N* (%)				
Young adults	539 (12.6)	407 (13.3)	132 (10.9)	0.058
Middle age	2440 (57.0)	1721 (56.0)	719 (59.3)	
Elderly	1305 (30.5)	943 (30.7)	362 (29.8)	
Sex, *N* (%)				
Male	2252 (52.6)	1528 (49.8)	724 (59.7)	<0.001
Female	2032 (47.4)	1543 (50.2)	489 (40.3)	
Smoking status, *N* (%)				
Never	2815 (67.2)	2033 (67.7)	782 (65.9)	0.053
Current	993 (23.7)	716 (23.9)	277 (23.3)	
Former	380 (9.1)	252 (8.4)	128 (10.8)	
Drinking status, *N* (%)				
Never	3189 (76.2)	2324 (77.4)	865 (72.9)	0.001
Current	750 (17.9)	520 (17.3)	230 (19.4)	
Former	249 (6.0)	157 (5.2)	92 (7.8)	
Family history of diabetes, *N* (%)
Yes	1462 (35.0)	1025 (34.2)	437 (36.9)	0.101
No	2717 (65.0)	1970 (65.8)	747 (63.1)	
Duration of diabetes, *N* (%)
<5 years	1436 (33.5)	1037 (33.8)	399 (32.9)	0.089
5–10 years	1475 (34.4)	1079 (35.1)	396 (32.7)	
>10 years	1373 (32.1)	955 (31.1)	418 (34.5)	
**Physical measurements**				
SBP, mmHg	136.42 ± 19.37	136.95 ± 19.58	135.09 ± 18.76	0.005
DBP, mmHg	80.88 ± 11.69	81.07 ± 11.56	80.41 ± 11.99	0.093
BMI, N (%)				
Underweight	143 (3.3)	96 (3.1)	47 (3.9)	<0.001
Normal weight	1728 (40.3)	1173 (38.2)	555 (45.8)	
Overweight	1761 (41.1)	1273 (41.5)	488 (40.2)	
Obese	652 (15.2)	529 (17.2)	123 (10.1)	
Waist-hip ratio	0.94 ± 0.07	0.94 ± 0.07	0.93 ± 0.06	<0.001
**Laboratory measurements**
Albumin, g/L	37.3 ± 3.9	37.2 ± 4.1	37.5 ± 3.5	0.011
Lipid profile, mmol/L				
Triglycerides	1.60 (1.10–2.38)	1.72 (1.18–2.58)	1.35 (0.96–2.02)	<0.001
Total cholesterol	4.38 (3.70–5.07)	4.47 (3.78–5.17)	4.19 (3.57–4.81)	<0.001
LDL-C	2.80 ± 0.90	2.84 ± 0.92	2.68 ± 0.84	<0.001
HDL-C	1.05 ± 0.29	1.04 ± 0.29	1.07 ± 0.28	0.002
GLU, mmol/L	7.87 (6.02–10.35)	8.08 (6.16–10.55)	7.30 (5.81–9.98)	<0.001
Glycemic control, *N* (%)
Good	768 (17.9)	509 (16.6)	259 (21.4)	<0.001
Poor	3516 (82.1)	2562 (83.4)	954 (78.6)	
HOMA2-IR	1.10 (0.76–1.65)	1.13 (0.78–1.68)	1.02 (0.71–1.52)	<0.001
Serum calcium, mmol/L	2.22 (2.16–2.30)	2.23 (2.16–2.30)	2.22 (2.17–2.29)	0.547
Serum phosphorus, mmol/L	1.03 ± 0.18	1.03 ± 0.19	1.02 ± 0.18	0.068
Serum uric acid status, *N* (%)				
Normal	3805 (88.8)	2706 (88.1)	1099 (90.6)	0.020
High	479 (11.2)	365 (11.9)	114 (9.4)	
Serum creatinine, mg/dL	0.71 (0.58–0.88)	0.70 (0.57–0.87)	0.73 (0.60–0.91)	0.003
eGFR, mL/min/1.73m^2^	134.38 ± 48.94	134.84 ± 49.24	133.22 ± 48.17	0.328
24HUALB, mg/day	14.90 (6.00–61.30)	16.17 (6.20–69.48)	12.72 (5.60–44.00)	<0.001
**Complications and comorbidities**
DR, *N* (%)	1222 (28.5)	908 (29.6)	314 (25.9)	0.016
DKD, *N* (%)	1207 (28.2)	904 (29.4)	303 (25.0)	0.003
DFU, *N* (%)	245 (5.7)	195 (6.3)	50 (4.1)	0.005
DPN, *N* (%)	2173 (50.7)	1543 (50.2)	630 (51.9)	0.318
Hypertension, *N* (%)	2315 (54.0)	1701 (55.4)	614 (50.6)	0.005
Dyslipidemia, *N* (%)	2770 (64.7)	2058 (67.0)	712 (58.7)	<0.001
Coronary heart disease, *N* (%)	823 (19.2)	617 (20.1)	206 (17.0)	0.020
Cerebrovascular disease, *N* (%)	556 (13.0)	382 (12.4)	174 (14.3)	0.095
**Medication**				
BPLT, *N* (%)	1791 (41.8)	1313 (42.8)	478 (39.4)	0.045
LLT, *N* (%)	3164 (73.9)	2315 (75.4)	849 (70.0)	<0.001
GLT, *N* (%)				
No medications	117 (2.7)	82 (2.7)	35 (2.9)	0.009
OHA only	1220 (28.5)	831 (27.1)	389 (32.1)	
Insulin only	828 (19.3)	601 (19.6)	227 (18.7)	
OHA plus insulin	2119 (49.5)	1557 (50.7)	562 (46.3)	

*VDD, vitamin D deficiency; SBP, systolic blood pressure; DBP, diastolic blood pressure; BMI, body mass index; HDL-C, high-density lipoprotein cholesterol; LDL-C, low-density lipoprotein cholesterol; GLU, fasting plasma glucose; HOMA2-IR, Homeostasis Model Assessment 2-insulin resistance; eGFR, estimated glomerular filtration rate; 24HUALB, 24-h urine albumin; DR, diabetic retinopathy; DKD, diabetic kidney disease; DFU, diabetic foot ulcers; DPN, diabetic peripheral neuropathy; BPLT, blood pressure lowering therapy; LLT, lipid lowering therapy; GLT, glucose lowering therapy; OHA, oral hypoglycemic agents*.

In bivariate analyses, compared with persons without VDD, persons with VDD were more likely to be female (50.2 vs. 40.3%, *P* < 0.001), never drunk (77.4 vs. 72.9%, *P* = 0.001), have higher systolic blood pressure (*P* = 0.005), higher rates of obesity (17.2 vs. 10.1%, *P* < 0.001) and higher waist-hip ratio (*P* < 0.001). With regard to laboratory measurements, higher triglycerides, total cholesterol, LDL-C, fasting plasma glucose, HOMA2-IR, and 24HUALB were observed in the VDD group (all *P* < 0.001), whereas relatively lower albumin, HDL-C, and serum creatinine were detected compared with the patients without VDD (all *P* < 0.05). Meanwhile, patients with VDD were more prone to have poor glycemic control (83.4 vs. 78.6%, *P* < 0.001) and higher level of serum uric acid (11.9 vs. 9.4%, *P* = 0.02). Additionally, participants with VDD were more vulnerable to DR (29.6 vs. 25.9%, *P* = 0.016), DKD (29.4 vs. 25.0%, *P* = 0.003), DFU (6.3 vs. 4.1%, *P* = 0.005), hypertension (55.4 vs. 50.6%, *P* = 0.005), dyslipidemia (67.0 vs. 58.7%, *P* < 0.001), and coronary heart disease (20.1 vs. 17.0%, *P* = 0.02), relative to those without VDD. Significant differences were also found with respect to medication between the two groups, including BPLT, LLT, and GLT (all *P* < 0.05). Besides, Supplementary Table 1 displays the prevalence of VDD and three diabetic vascular complications (i.e., DR, DKD, and DFU). Overall, the prevalence of VDD accounted to 71.7% (95% confidence intervals [CI]: 70.3–73.0%), which was defined as 25(OH)D levels <50 nmol/L. The prevalence of DR and DKD were very similar at 28.5% (95% CI: 27.2–29.9%) and 28.2% (95% CI: 26.8–29.5%), respectively. Besides, a total of 5.7% (95% CI: 5.1–6.5%) of patients had a diagnosis of DFU in this study.

### Association Between Prevalence of DR and VDD Status

**Figure 1:**
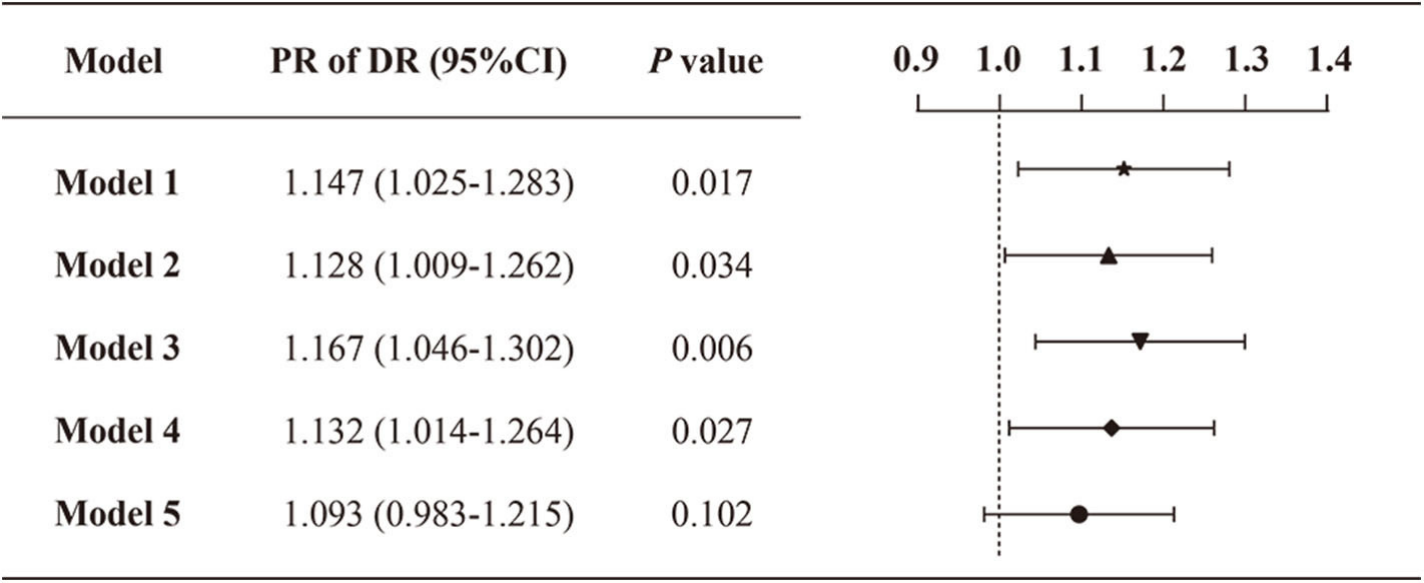
Prevalence ratios of DR by VDD status among the study population. Model 1: crude, unadjusted; model 2: adjusted for age, sex; model 3: adjusted for age, sex, duration of diabetes, smoking status, drinking status, BMI, and waist-hip ratio; model 4: model 3 + albumin, triglycerides, HDL-C, glycemic control, HOMA2-IR, serum calcium, serum phosphorus, serum uric acid, serum creatine, 24HUALB; model 5: model 4 + diabetic complications (DKD, DFU, DPN), related comorbidities (coronary heart disease, cerebrovascular disease, and hypertension), and medications (BPLT, LLT and GLT).

Figure 1 presents the PR and 95% CI for DR by VDD status. In unadjusted analyses (model 1), DR was associated with VDD status (PR: 1.147; 95% CI: 1.025–1.283). The association was retained when adjusting for age and sex (model 2). Meanwhile, a slightly larger PR was observed when adjusting for other demographics and physical measurements besides age and sex (model 3). Further adjusting for laboratory measurements other than diabetic complications, related comorbidities, and medications attenuated the risk, although the association remained significant (model 4) (PR: 1.132; 95% CI: 1.014–1.264). However, the significance diminished after adjusting for all variables in the final adjusted model (model 5) (PR: 1.093; 95% CI: 0.983–1.215). The final adjusted model is displayed in detail in Supplementary Table 2.

### Association Between Prevalence of DKD and VDD Status

**Figure 2:**
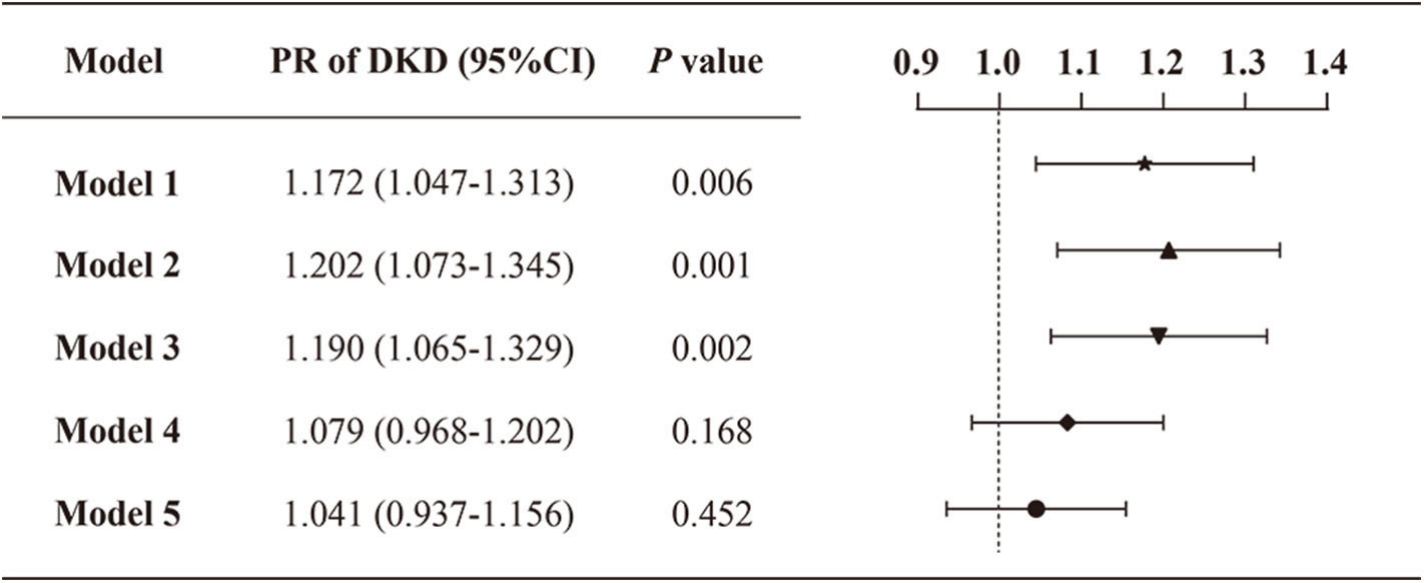
Prevalence ratios of DKD by VDD status among the study population. Model 1: crude, unadjusted; model 2: adjusted for age, sex; model 3: adjusted for age, sex, duration of diabetes, smoking status, drinking status, BMI and waist-hip ratio; model 4: model 3 + albumin, total cholesterol, LDL-C, glycemic control, HOMA2-IR, serum calcium, serum phosphorus, serum uric acid, serum creatine, 24HUALB; model 5: model 4 + diabetic complications (DR, DFU, DPN), related comorbidities (coronary heart disease, cerebrovascular disease, and hypertension), and medications (BPLT, LLT, and GLT).

Models 1–5 in Figure 2 present the Poisson regression with robust variance models for the assessment of the correlation between VDD status and the prevalence of DKD. The prevalence of DKD was significantly higher in the VDD group in comparison to no-VDD persons in the crude analysis (model 1) (PR: 1.172; 95% CI: 1.047–1.313). The associations remained markedly significant when adjusting for age and sex only (model 2) (PR: 1.202; 95% CI: 1.073–1.345) or additionally adjusting other demographics and physical measurements (model 3) (PR: 1.190; 95% CI: 1.065–1.329). However, when other possible explanatory variables associated with DKD in univariate analysis were considered, including laboratory factors, diabetic complications, related comorbidities and medications, no significant association between the VDD status and prevalence of DR was demonstrated (model 4–5). Model 5 adjusting for all variables is displayed in detail in Supplementary Table 3.

### Association Between DFU and VDD Status

**Figure 3:**
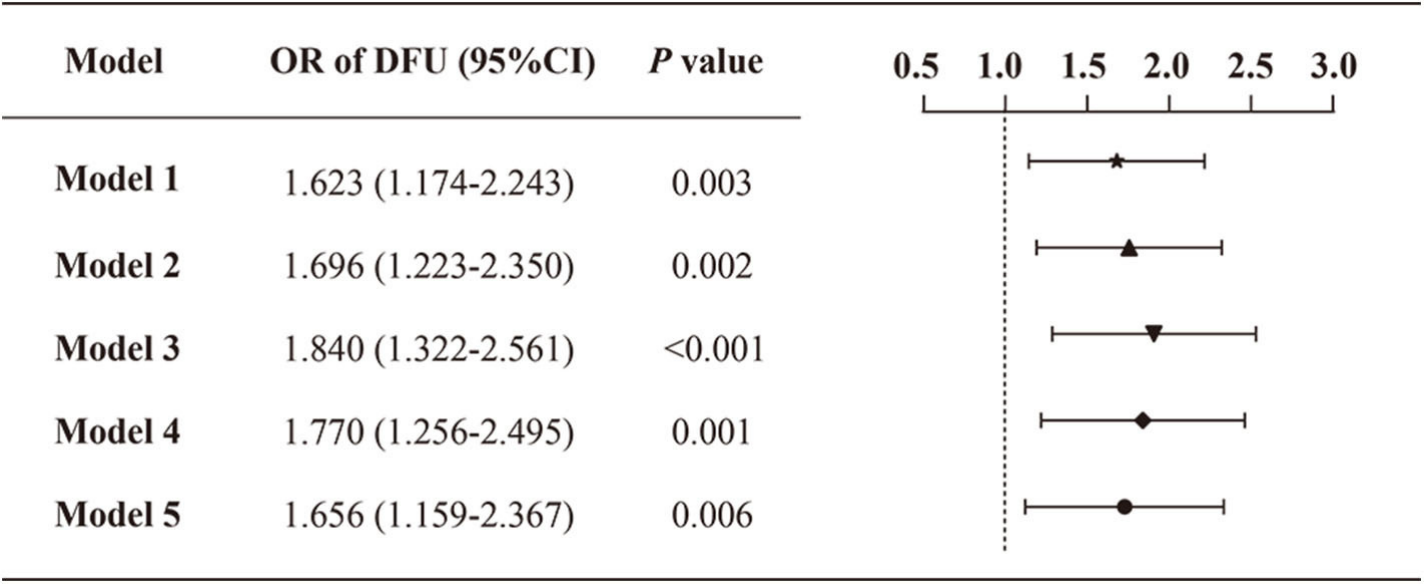
Odds ratio of DFU by VDD status among the study population. Model 1: crude, unadjusted; model 2: adjusted for age, sex; model 3: adjusted for age, sex, duration of diabetes, smoking status, drinking status, family history of diabetes, BMI and waist-hip ratio; model 4: model 3 + albumin, triglycerides, total cholesterol, HDL-C, LDL-C, serum calcium, serum creatinine, 24HUALB; model 5: model 4 + diabetic complications (DR, DKD, DPN), related comorbidities (coronary heart disease, cerebrovascular disease, and hypertension), and medications (BPLT, LLT and GLT).

As the prevalence of DFU was not common (5.7%) in the study population, we next performed logistic regression analyses to assess the relationship between VDD status and the prevalence of DFU (Figure 3). In the crude model (model 1), the presence of VDD was associated with an increased prevalence of DFU (odds ratio [OR]: 1.623; 95% CI: 1.174–2.243). The association was slightly enhanced when adjusting age and sex (model 2) (OR: 1.696; 95% CI: 1.223–2.350). Besides, an obvious increase in the odds of DFU was observed after additional adjustment of other demographics and physical measurements (i.e., duration of diabetes, smoking status, drinking status, family history of diabetes, BMI, and waist-hip ratio) (model 3) (OR: 1.840; 95% CI: 1.322–2.561). When further controlling for the biochemical indices (i.e., albumin, triglycerides, total cholesterol, HDL-C, LDL-C, serum calcium, serum creatine, and 24HUALB) (model 4), participants with VDD still had a greater prevalence of DFU compared with the no-VDD group. Final adjustment for diabetic complications, related comorbidities and medications, attenuated the association between VDD status and DFU, but did not remove statistical significance (model 5) (OR: 1.656; 95% CI: 1.159–2.367). Supplementary Table 4 displays all variables included in the final adjusted model.

### Vitamin D Metrics by DFU Status, Overall and by Sex

We also evaluated the proportions of VDD and 25(OH) levels between the DFU group and the no-DFU group (Table 2). Overall, compared with subjects without DFU, persons with DFU had higher prevalence of VDD (79.59 vs. 71.21%, *P* = 0.005) and lower serum 25(OH)D levels (36.96 ± 18.03 nmol/L vs. 40.97 ± 17.82 nmol/L, *P* = 0.001). When stratified by sex, similar results were observed in men (all *P* < 0.05), while there were no significant differences in the prevalence of VDD between these two groups in women (83.02 vs. 75.55%, *P* = 0.08). Moreover, in the DFU group, it seemed that the 25(OH)D levels in men were slightly greater than that in women, although it did not reach statistical significance (38.86 ± 19.26 nmol/L vs. 34.46 ± 16.03 nmol/L, *P* = 0.058).

**Table 2:** Vitamin D metrics by DFU status, overall and by sex.

	**DFU**	**No DFU**	***P* value**
**Overall**			
*N*	245	4039	
VDD, *N* (%)	195 (79.59)	2876 (71.21)	0.005
25(OH)D, nmol/L	36.96 ± 18.03	40.97 ± 17.82	0.001
**Male**			
*N*	139	2113	
VDD, *N* (%)	107 (76.98)	1421 (67.25)	0.017
25(OH)D, nmol/L	38.86 ± 19.26	42.87 ± 18.04	0.012
**Female**			
*N*	106	1926	
VDD, *N* (%)	88 (83.02)	1455 (75.55)	0.080
25(OH)D, nmol/L	34.46 ± 16.03	38.89 ±17.36	0.010

*VDD, vitamin D deficiency; DFU, diabetic foot ulcer; 25(OH)D, 25-hydroxyvitamin D*.

## Discussion

In this study, ~71.7% of Chinese hospitalized patients with T2DM developed VDD. Patients with VDD had higher prevalence of DFU after adjustment for demographics, physical measurements, laboratory indices, related treatment factors, and comorbidities compared with patients without VDD, whereas the associations between VDD status and another two microvascular complications (i.e., DR and DKD) were not statistically significant.

VDD is a growing epidemic condition around the world (32), the prevalence of which varies by race, latitudes, and seasons (18). An estimated 50–80% of the general population is affected by vitamin D insufficiency or VDD globally (33). In northwest and north China, the prevalence of VDD was about 75.2 and 87.1%, respectively (34, 35). VDD is also quite common among Chinese patients with T2DM. A Chinese cross-sectional survey among diabetic inpatients reported that the proportions of persons with VDD were 83.5%, the recruitment center of which was located in north China (latitude 34°-37° N) (36). Besides, ~62.7% of T2DM subjects were affected in two epidemiological studies conducted in Nanjing, which is located in eastern coastal China (latitude 31°-33° N) (19). In present study, we found that the prevalence of VDD was about 71.7% among this study population with T2DM, who were recruited in Changsha, a city located in central China (latitude 27°-29° N). Although the discordance in prevalence of VDD could partially explained by latitude, other factors such as diet and lifestyle must be considered.

Furthermore, we found that the prevalence of DR and DKD in this study population was 28.5 and 28.2%, respectively. The results were roughly in line with previous studies (37, 38). However, the proportion of DFU (5.7%, 95% CI: 5.1–6.5%) was much lower in comparison to a previous study conducted in Wuhan, China (11.4%) (39). We believed that the actual prevalence of DFU may be underestimated. One plausible interpretation was the presence of missed diagnosis of DFU during admission. Some patients without acute symptoms (e.g., ulceration, infection, swollen foot with pain) may not receive further examinations due to socioeconomic concerns. Besides, DFU is generally considered as the consequences of diabetic neuropathy and/or peripheral arterial disease (7). Sometimes patients with peripheral arterial disease may remain undiagnosed until severe tissue loss appears, which also add to the difficulty of the correct diagnosis of DFU (40). We also addressed that in rural areas of China, a higher proportion of DFU remains undiagnosed because of the less medical access and limited knowledge on this severe diabetic complication. Therefore, future efforts should be directed at early diagnosis of DFU in both urban and rural areas.

DR and DKD are common microvascular complications of diabetes (7). Many clinical studies have recognized VDD as the risk factor for DR (16, 25, 41) and DKD (17, 42), whereas other epidemiological researches showed opposite results (21, 22, 43). In this study, we revealed that the correlation between VDD status and the prevalence of DR or DKD was not statistically significant after adjusting laboratory measurements, diabetic complications, related comorbidities, and medications besides the adjustment for demographics and physical measurements. These conflict results may be mainly due to the differences in study population and covariates included in the regression analyses. Considering all the published data, the connection between vitamin D levels and risk of DR or DKD remains inconclusive in Chinese population and further studies are required.

DFU, a complex and costly complication of diabetes, is associated with other severe conditions such as peripheral neuropathy, peripheral vascular disease, secondary infections and it can lead to lower extremity amputation (44). Until now, there were only two studies from India revealed that low vitamin D may play a critical role in the pathogenesis of DFU (45, 46). We investigated the association between VDD and risk of DFU in Chinese T2DM patients for the first time. In accord with these studies (45, 46), we demonstrated that there were significant 65.6% higher odds of DFU for those participants with VDD than those without VDD when adjusting all potential confounders collected in this study, including demographics, physical measurements, biochemical indices, related complications, and comorbidities, as well as medications. However, Afarideh et al. (23) reported no difference in vitamin D levels in Iranian patients with DFU compared with diabetic patients without DFU. Regardless of this contradiction, many lines of evidence supported the favorable effects of vitamin D on DFU, especially on wound healing, which is impaired in diabetic patients due to persistent inflammation (47). Vitamin D is essential in maintaining the normal immune system (48). Vitamin D could suppress T cell proliferation and inhibit the secretion of T helper type 1 cytokines (e.g., interferon-γ and interleukin-2), while augmenting the production of T helper type 2 cytokines (49), thereby accelerating wound healing. Another study found that calcitriol, the most active vitamin D metabolite, not only augmented proangiogenic factors in keratinocytes but also induced antimicrobial peptides expression in a DFU model (50). Besides, vitamin D may improve wound healing by suppressing endoplasmic reticulum stress (51), oxidative stress (52), and the NF-κB-mediated inflammatory gene expression (47). More importantly, vitamin D signaling may be involved in the proliferation, migration, and differentiation of epidermal stem cells and progeny during cutaneous wound repair (53). Despite the abundance of preclinical data regarding vitamin D and wound healing, only one randomized controlled trial that evaluated the effects of vitamin D supplementation on DFU patients has been reported (52). Razzaghi et al. (52) demonstrated that vitamin D supplementation for 12 weeks resulted in a significant improvement on wound evolution, including ulcer length, width, depth, and erythema rate. In the light of current evidence, we proposed that VDD may be associated with a higher prevalence of DFU and vitamin D supplementation may be a potential therapeutic option for DFU patients with low vitamin D levels, although whether VDD is the cause or the result of DFU remains unknown.

Additionally, previous studies have revealed that patients with DFU had higher prevalence of VDD and lower vitamin D levels in comparison to diabetic patients without DFU (17, 20, 45, 54, 55). We reported that the prevalence of VDD in participants with DFU was about 80%. Meanwhile, the prevalence of VDD in male and female patients with DFU was ~77 and 83%, respectively. One explanation for these results could be their immobilization caused by DFU, thus leading to less outdoor activities and sunlight exposure.

To our surprise, the differences in the prevalence of DPN in patients with and without VDD was not statistically significant in this study, although DPN has been associated with VDD in previous studies (56, 57). However, there is still a small amount of literature reported different results. For example, a cross-sectional study that involved 239 participants with T2DM revealed that neuropathic pain was not associated with serum vitamin D (58). Besides, a case-control study included 25 T2DM patients with DPN and 25 healthy controls reported that the severe form of neuropathy was more liable for lower vitamin D levels (59). Meanwhile, another study showed that approximately 60% of DFU are primarily neuropathic in origin (60). These data suggest that the correlation between DFU and VDD may not reflect the relationship between DPN and VDD. Besides large population sampling and different neuropathy assessment tools, another explanation for the low differences in DPN between patients with and without VDD is that some patients (e.g., patients with poor economic conditions) with DPN may not be able to accept related examinations and get timely diagnosis. Because of the potential correlation between DPN and DFU, we included DPN as a covariate both in univariate logistic regression analyses (*P* value < 0.001, data not shown) and multivariable model (Supplementary Table 4; OR: 2.967; 95% CI: 2.066–4.261). Thus, we have adjusted the effects of DPN when exploring the association between VDD and DFU. To further investigate the association between DPN and VDD, more detailed data about DPN (e.g., the severity of DPN) will be collected in future studies.

The results of our study have several clinical implications for healthcare delivery. We demonstrated for the first time that low vitamin D was associated with higher prevalence of DFU in Chinese T2DM population. We highlighted that vitamin D level may be a modifiable factor in the prevention of diabetic foot complications. Besides, our findings extend the knowledge about the correlation between VDD and DR/DKD, although the statistical significance was removed in the final adjusted model. Finally, considering the high prevalence of VDD, we proposed that screening 25(OH)D levels may be beneficial for patients with diabetes. It not only incites patients to change lifestyles and dietary timely so as to increase the levels of vitamin D, but also help minimize the occurrence of complications, and improve the quality of life.

Our study has some important strengths, including the relatively large sample size, a well-defined study population, the availability of multiple covariates, and strong quality control. Notwithstanding these strengths, some limitations should be considered. First, the study design was cross-sectional, thus the temporality of this association between VDD status and DFU cannot be confirmed. Second, the current study was a single-center study, thus our results may not be generalizable to the entire Chinese T2DM population. Third, other potential confounders, including diet, sunlight exposure, physical activity, economic status, season of vitamin D detection, supplementation of vitamin D (by food or drugs) were not available in the analysis. To clarify the associations between vitamin D levels and diabetes-related complications and to assess the benefits of vitamin D supplementation, multi-center randomized controlled trials and larger-scale prospective studies are required.

## Conclusion

VDD is a very common condition among Chinese T2DM patients. Different from DR and DKD, the association between VDD status and DFU was still significant after adjusting numerous potential confounders, including demographics, physical measurements, biochemical indices, related comorbidities and complications, as well as medication use. Vitamin D supplementation by dietary or other intervention strategies to correct VDD in Chinese diabetic population may help prevent the development of DFU.

## Data Availability Statement

The raw data supporting the conclusions of this article will be made available by the authors, without undue reservation.

## Ethics Statement

The studies involving human participants were reviewed and approved by the ethics committee of the Second Xiangya Hospital of Central South University. Written informed consent for participation was not required for this study in accordance with the national legislation and the institutional requirements.

## Author Contributions

YX designed the study, analyzed the data, interpreted the results, and drafted the manuscript. YX, LW, and XX contributed to data collection and manuscript revision. MY provided support for interpreting the results and revising the manuscript. LS is the corresponding author and was involved in the study design, data interpretation and manuscript revision. All authors read and approved the final manuscript.

## Conflict of Interest

The authors declare that the research was conducted in the absence of any commercial or financial relationships that could be construed as a potential conflict of interest.
