# Supplementary material for: Association Between Vitamin D Status and Diabetic Complications in Patients With Type 2 Diabetes Mellitus: A Cross-Sectional Study in Hunan China
Source: Front Endocrinol (Lausanne). 2020 Sep 16;11:564738. doi: 10.3389/fendo.2020.564738 (PMC7525149; doi:10.3389/fendo.2020.564738)
Supplement: Supplementary file 1 [file Table_1.DOCX]

**Supplementary Table 1** **|** Prevalence of VDD and three vascular complications of T2DM among the study population.

|  | **Prevalence (%)** | **95% CI (%)** |
| --- | --- | --- |
| VDD | 71.7 | 70.3-73.0 |
| DR | 28.5 | 27.2-29.9 |
| DKD | 28.2 | 26.8-29.5 |
| DFU | 5.7 | 5.1-6.5 |

T2DM, type 2 diabetes mellitus; VDD, vitamin D deficiency; DR, diabetic retinopathy; DKD, diabetic kidney disease; DFU, diabetic foot ulcer; CI, confidence intervals.

**Supplementary Table 2 |** Multivariable regression analysis relating the association of VDD status to DR.

| **Variable** | **PR (95% CI)** | ***P* value** |
| --- | --- | --- |
| VDD | 1.093 (0.983-1.215) | 0.102 |
| Age |  |  |
| Young adults | Referent |  |
| Middle age | 1.045 (0.865-1.263) | 0.646 |
| Elderly | 0.772 (0.625-0.954) | 0.017 |
| Sex (male) | 0.776 (0.681-0.885) | 0.000 |
| Smoking status |  |  |
| Never | Referent |  |
| Current | 0.987 (0.846-1.151) | 0.886 |
| Former | 0.947 (0.772-1.163) | 0.606 |
| Drinking status |  |  |
| Never | Referent |  |
| Current | 0.896 (0.765-1.049) | 0.173 |
| Former | 1.022 (0.820-1.273) | 0.849 |
| Duration of Diabetes |  |  |
| <5 years | Referent |  |
| 5-10 years | 1.323 (1.139-1.536) | <0.001 |
| >10 years | 1.804 (1.551-2.098) | <0.001 |
| BMI |  |  |
| Underweight | 0.916 (0.720-1.165) | 0.475 |
| Normal weight | Referent |  |
| Overweight | 1.034 (0.933-1.147) | 0.522 |
| Obese | 0.929 (0.796-1.083) | 0.345 |
| Waist-hip ratio | 0.902 (0.418-1.948) | 0.793 |
| Albumin | 0.992 (0.980-1.005) | 0.236 |
| Triglycerides | 0.976 (0.955-0.997) | 0.027 |
| HDL-C | 0.991 (0.854-1.150) | 0.903 |
| Glycemic control |  |  |
| Good | Referent |  |
| Poor | 1.079 (0.947-1.229) | 0.255 |
| HOMA2-IR | 0.962 (0.906-1.021) | 0.203 |
| Serum calcium | 0.855 (0.528-1.385) | 0.524 |
| Serum phosphorus | 1.356 (1.064-1.728) | 0.014 |
| Serum uric acid status |  |  |
| Normal | Referent |  |
| High | 1.027 (0.889-1.188) | 0.715 |
| Serum creatinine | 1.212 (1.091-1.347) | <0.001 |
| 24HUALB | 1.000 (1.000-1.000) | 0.747 |
| DKD | 1.537 (1.382-1.710) | <0.001 |

**Supplementary Table 2 |** (continued).

| **Variable** | **PR (95% CI)** | ***P* value** |
| --- | --- | --- |
| DFU | 1.184 (1.026-1.367) | 0.021 |
| DPN | 2.171 (1.909-2.469) | <0.001 |
| Coronary heart disease | 1.023 (0.918-1.140) | 0.676 |
| Cerebrovascular disease | 1.134 (1.002-1.282) | 0.046 |
| Hypertension | 1.013 (0.893-1.149) | 0.841 |
| BPLT | 1.180 (1.050-1.327) | 0.006 |
| LLT | 0.976 (0.871-1.092) | 0.668 |
| GLT |  |  |
| No medications | Referent |  |
| OHA only | 1.266 (0.780-2.056) | 0.340 |
| Insulin only | 1.795 (1.108-2.909) | 0.018 |
| OHA plus insulin | 1.747 (1.082-2.821) | 0.023 |

VDD, vitamin D deficiency; DR, diabetic retinopathy; BMI, body mass index; HDL-C, high-density lipoprotein cholesterol; HOMA2-IR, Homeostasis Model Assessment 2-insulin resistance; 24HUALB, 24-hour urine albumin; DKD, diabetic kidney disease; DFU, diabetic foot ulcers; DPN, diabetic peripheral neuropathy; BPLT, blood pressure lowering therapy; LLT, lipid lowering therapy; GLT, glucose lowering therapy; OHA, oral hypoglycemic agents; PR, prevalence ratios; CI, confidence intervals.

**Supplementary Table 3 |** Multivariable regression analysis relating the association of VDD status to DKD.

| **Variable** | **PR (95% CI)** | ***P* value** |
| --- | --- | --- |
| VDD | 1.041 (0.937-1.156) | 0.452 |
| Age |  |  |
| Young adults | Referent |  |
| Middle age | 0.870 (0.735-1.030) | 0.106 |
| Elderly | 0.873 (0.724-1.053) | 0.156 |
| Sex (male) | 1.129 (0.999-1.276) | 0.052 |
| Smoking status |  |  |
| Never | Referent |  |
| Current | 1.050 (0.914-1.207) | 0.492 |
| Former | 1.050 (0.885-1.245) | 0.578 |
| Drinking status |  |  |
| Never | Referent |  |
| Current | 0.884 (0.769-1.016) | 0.082 |
| Former | 0.932 (0.767-1.133) | 0.479 |
| Duration of Diabetes |  |  |
| <5 years | Referent |  |
| 5-10 years | 1.343 (1.167-1.546) | <0.001 |
| >10 years | 1.519 (1.314-1.757) | <0.001 |
| BMI |  |  |
| Underweight | 1.076 (0.826-1.401) | 0.587 |
| Normal weight | Referent |  |
| Overweight | 1.043 (0.941-1.156) | 0.426 |
| Obese | 1.053 (0.916-1.211) | 0.468 |
| Waist-hip ratio | 2.420 (1.157-5.060) | 0.019 |
| Albumin | 0.973 (0.961-0.985) | <0.001 |
| Total cholesterol | 1.044 (0.980-1.112) | 0.185 |
| LDL-C | 0.973 (0.898-1.055) | 0.509 |
| Glycemic control |  |  |
| Good | Referent |  |
| Poor | 1.070 (0.947-1.209) | 0.278 |
| HOMA2-IR | 1.007 (0.966-1.050) | 0.747 |
| Serum Calcium | 0.914 (0.560-1.491) | 0.719 |
| Serum phosphorus | 0.989 (0.777-1.259) | 0.926 |
| Serum uric acid status |  |  |
| Normal | Referent |  |
| High | 1.087 (0.960-1.231) | 0.187 |
| Serum creatinine | 1.682 (1.535-1.844) | <0.001 |
| 24HUALB | 1.000 (1.000-1.000) | 0.004 |
| DR | 1.471 (1.336-1.620) | <0.001 |

**Supplementary Table 3 |** (continued).

| **Variable** | **PR (95% CI)** | ***P* value** |
| --- | --- | --- |
| DFU | 1.198 (1.023-1.404) | 0.025 |
| DPN | 1.258 (1.134-1.396) | <0.001 |
| Coronary heart disease | 0.970 (0.873-1.076) | 0.561 |
| Cerebrovascular disease | 1.091 (0.972-1.226) | 0.141 |
| Hypertension | 1.049 (0.914-1.204) | 0.494 |
| BPLT | 1.962 (1.732-2.222) | <0.001 |
| LLT | 1.214 (1.079-1.366) | 0.001 |
| GLT |  |  |
| No medications | Referent |  |
| OHA only | 0.901 (0.628-1.293) | 0.572 |
| Insulin only | 1.105 (0.770-1.587) | 0.588 |
| OHA plus insulin | 0.987 (0.690-1.410) | 0.941 |

VDD, vitamin D deficiency; DKD, diabetic kidney disease; BMI, body mass index; LDL-C, low-density lipoprotein cholesterol; HOMA2-IR, Homeostasis Model Assessment 2-insulin resistance; 24HUALB, 24-hour urine albumin; DR, diabetic retinopathy; DFU, diabetic foot ulcers; DPN, diabetic peripheral neuropathy; BPLT, blood pressure lowering therapy; LLT, lipid lowering therapy; GLT, glucose lowering therapy; OHA, oral hypoglycemic agents; PR, prevalence ratios; CI, confidence intervals.

**Supplementary Table 4 |** Multivariable regression analysis relating the association of VDD status to DFU.

| **Variable** | **OR (95% CI)** | ***P* value** |
| --- | --- | --- |
| VDD | 1.656 (1.159-2.367) | 0.006 |
| Age |  |  |
| Young adults | Referent |  |
| Middle age | 0.785 (0.446-1.381) | 0.401 |
| Elderly | 1.374 (0.748-2.527) | 0.306 |
| Sex (male) | 1.368 (0.913-2.049) | 0.128 |
| Smoking status |  |  |
| Never | Referent |  |
| Current | 0.986 (0.629-1.547) | 0.952 |
| Former | 0.924 (0.524-1.629) | 0.784 |
| Drinking status |  |  |
| Never | Referent |  |
| Current | 1.101 (0.707-1.716) | 0.670 |
| Former | 1.344 (0.744-2.428) | 0.327 |
| Family history of diabetes | 1.626 (1.211-2.184) | 0.001 |
| Duration of diabetes |  |  |
| <5 years | Referent |  |
| 5-10 years | 0.770 (0.524-1.131) | 0.183 |
| >10 years | 0.616 (0.409-0.928) | 0.020 |
| BMI |  |  |
| Underweight | 1.776 (0.975-3.234) | 0.060 |
| Normal weight | Referent |  |
| Overweight | 0.816 (0.588-1.132) | 0.223 |
| Obese | 0.573 (0.328-0.999) | 0.050 |
| Waist-hip ratio | 0.646 (0.058-7.262) | 0.724 |
| Albumin | 0.864 (0.831-0.899) | <0.001 |
| Triglycerides | 0.831 (0.698-0.988) | 0.036 |
| Total cholesterol | 0.952 (0.559-1.619) | 0.855 |
| LDL-C | 0.909 (0.532-1.552) | 0.726 |
| HDL-C | 0.387 (0.170-0.880) | 0.024 |
| Serum calcium | 1.303 (0.300-5.657) | 0.724 |
| Serum creatine | 0.621 (0.424-0.908) | 0.014 |
| 24HUALB | 1.000 (1.000-1.000) | 0.462 |
| DR | 1.479 (1.084-2.019) | 0.014 |
| DKD | 1.705 (1.214-2.396) | 0.002 |
| DPN | 2.967 (2.066-4.261) | <0.001 |
| Coronary heart disease | 0.989 (0.693-1.413) | 0.953 |
| Cerebrovascular disease | 0.990 (0.669-1.465) | 0.959 |
| Hypertension | 1.582 (1.084-2.309) | 0.017 |
| BPLT | 0.787 (0.551-1.123) | 0.187 |

**Supplementary Table 4 |** (continued).

| **Variable** | **OR (95% CI)** | ***P* value** |
| --- | --- | --- |
| LLT | 2.274 (1.529-3.382) | <0.001 |
| GLT |  |  |
| No medications | Referent |  |
| OHA only | 0.364 (0.144-0.921) | 0.033 |
| Insulin only | 1.171 (0.493-2.780) | 0.720 |
| OHA plus insulin | 0.736 (0.312-1.734) | 0.483 |

VDD, vitamin D deficiency; DFU, diabetic foot ulcers; BMI, body mass index; HDL-C, high-density lipoprotein cholesterol; LDL-C, low-density lipoprotein cholesterol; 24HUALB, 24-hour urine albumin; DR, diabetic retinopathy; DKD, diabetic kidney disease; DPN, diabetic peripheral neuropathy; BPLT, blood pressure lowering therapy; LLT, lipid lowering therapy; GLT, glucose lowering therapy; OHA, oral hypoglycemic agents; OR, odds ratio; CI, confidence intervals.
